# Identification and validation of GABA‐driven subtypes and prognosis signature of lung adenocarcinoma

**DOI:** 10.1002/ctm2.1450

**Published:** 2023-10-19

**Authors:** Lifeng Li, Shilong Sun, Mengle Peng, Kai Wu, Xiaoran Duan, Ruyue Xue, Meijia Yang, Xu Zhang, Jie Zhao

**Affiliations:** ^1^ Cancer Center The First Affiliated Hospital of Zhengzhou University Zhengzhou Henan China; ^2^ National Engineering Laboratory for Internet Medical Systems and Applications The First Affiliated Hospital of Zhengzhou University Zhengzhou Henan China; ^3^ Medical school Huanghe Science and Technology University Zhengzhou Henan China; ^4^ Department of Pharmacy The First Affiliated Hospital of Zhengzhou University Zhengzhou Henan China; ^5^ Department of Clinical Laboratory Henan No. 3 Provincial People's Hospital Zhengzhou Henan China; ^6^ Department of Thoracic Surgery The First Affiliated Hospital of Zhengzhou University Zhengzhou Henan China

Dear Editor,

Recent studies confirmed that the inhibitory neurotransmitter gamma‐aminobutyric acid (GABA) can regulate the proliferation and migration of tumour cells.^1^, ^2^, ^3^, ^4^ Lung adenocarcinoma (LUAD) is the subtype with the largest proportion of lung cancer.^5^, ^6^ Huang et al. confirmed that GABA content in LUAD tissues was obviously higher than normal tissues by collecting clinical tissues.^7^ By analysing GABA expression in different LUAD patients, we established a GABA‐driven LUAD classification and developed a GABA‐driven prognostic signature. We demonstrated that GABA can be used as a biomarker for prognostic assessment and individualised treatment of LUAD.

We first established a GABA‐driven LUAD classification using conventional genetic databases and clustering algorithms. By performing an overall search of GABA‐related pathways from the Molecular Signatures Database (MSigDB),^8^ we acquired 221 GABA‐related genes from 15 gene sets, including Gene Ontology (GO), Human Phenotype Ontology (HPO), WikiPathways, Biocarta, Reactome and many other diverse biological functions and sources. After difference analysis of these genes in tumour and normal tissue by ‘limma’ package, 108 GABA core difference genes were obtained according to the absolute value of logFC > .5 (Table S1). Based on 108 GABA core differential genes, consensus clustering was performed in The Cancer Genome Atlas (TCGA)‐LUAD queue (*n* = 555) by using the ‘ConsensusClusterPlus’ package. *K* value of 4 was selected by the delta area plot, then we classified LUAD patients into four subtypes (Figure 1A and B). We further explored the expression of GABA core genes in four subtypes (Figure S1). The key genes for GABA include the synthetic rate‐limiting enzymes glutamic acid decarboxylase1 (GAD1) and GAD2 and the receptors GABAA and GABAB. Kaplan–Meier analysis of four subtypes showed that cluster 1 had the best prognosis (Figure 1C) (*p* < .0001). And cluster 2 patients showed a significantly worse prognosis compared with non‐cluster 2 patients (Figure S2A‐D) (*p* < .0001). Differential analysis was conducted in the TCGA cohort using the ‘limma’ package, and 500 characteristic genes for each subtype were obtained, with a total of 2000 (Table S2). Based on the characteristic genes of four subtypes, the samples were classified in three Gene Expression Omnibus (GEO) queues (GSE31210, GSE41271, GSE72094) using Nearest Template Prediction (NTP) algorithm.^9^ We then confirmed the accuracy and stability of our TCGA‐LUAD classification by performing a survival analysis on the three GEO cohorts (Figure 1D‐F) and comparing the similarity between the training set TCGA and the three GEO validation sets using the SubMap algorithm (Figure 1G‐I). We selected the gene that contributed most to the prognosis of cluster 2, EZH2, and detected its expression in LUAD tissues. The results of immunohistochemistry (IHC) showed that EZH2 expression was increased in LUAD tissues of patients with poor prognosis (Figure 1J), which was consistent with our analysis results and further proved the accuracy of our LUAD classification based on the core differential genes.

**FIGURE 1:**
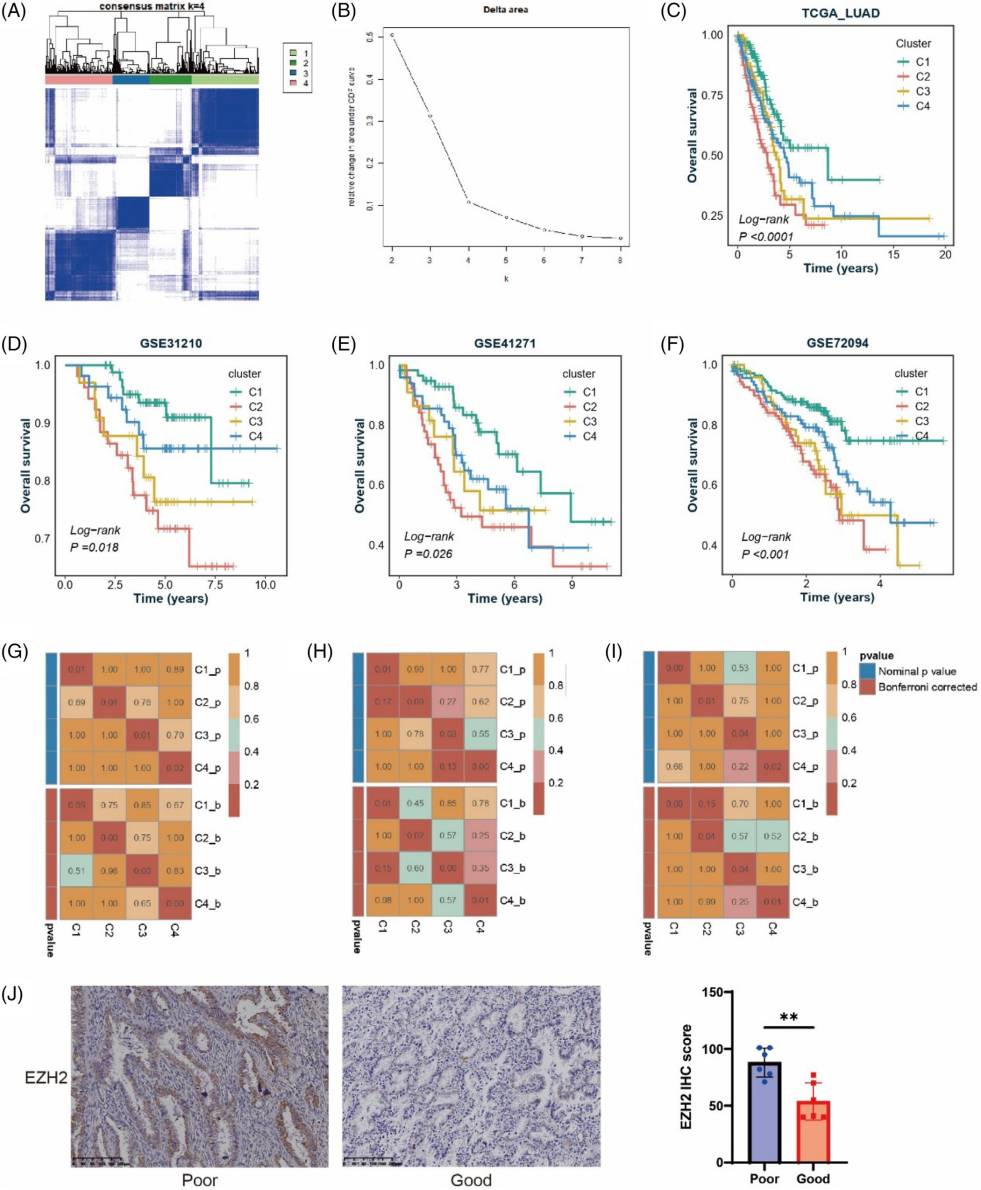
Construction and verification of GABA‐driven LUAD subtypes. (A) Consensus cluster analysis was performed on the TCGA‐LUAD queue. (B) Relative changes in the area under the cumulative distribution function (CDF) curve for *k* = 2–8. (C) Overall survival (OS) of patients in four clusters. (D–F) OS of each cluster in three validation cohorts. (G–I) SubMap analysis verified the similarity between TCGA queue and GEO queue. G to I correspond to GSE31210, GSE41271 and GSE72094, respectively. (J) EZH2 IHC and IHC scores of patients with good (*n* = 6) and poor prognosis (*n* = 6).

In order to study the potential biological functions of different subtypes, 9570 gene set pathways were collected from the MSigDB database, including 50 cancer hallmark gene sets, 186 Kyoto Encyclopedia of Genes and Genomes (KEGG) gene sets, 196 Pathway Interaction Database (PID) gene sets, 289 Biocarta gene sets, 1499 Reactome gene sets, and 7350 GO biological process gene sets. Function enrichment analysis was then conducted by using the ‘clusterProfiler’ R package. The results showed that cluster 1 was mainly enriched in pathways such as ‘immune response’, ‘T cells’ and ‘leukocyte’ (Figure S3A), which related to immunotherapy. The functions of the remaining subtypes are mainly concentrated in pathways related to cell proliferation, differentiation or energy metabolism (Figure S3B‐D). Considering the importance of immunotherapy in LUAD, we evaluated the immune landscape of each subtype. First, the degree of infiltration of some tumour immune cells in TCGA‐LUAD queue was assessed by the single‐sample gene set enrichment analysis (ssGSEA) algorithm. As shown in the thermal and box diagrams (Figure 2A), the immune cell infiltration of cluster 1 was obvious, covering CD8+T cells and B cells. Next, we evaluated the expression of each immune checkpoint in four subtypes (Figure 2B). It can be clearly observed that the expression of various immune checkpoints in cluster 1 were obviously higher than that in other subtypes, such as cytotoxic T lymphocyte antigen 4 (CTLA4). To further understand the immunotherapy status of various subtypes, tumour inflammation signature (TIS), antigen presentation score (APS), tumour immune dysfunction and exclusion (TIDE) methods were applied to evaluate the effectiveness of different subtypes to immunotherapy. As expected, cluster 1 showed higher scores in the three indicators (Figure 2C‐F) (TIS *p* = 4.9e‐06; APS *p* = 2.4e‐06; TIDE *p* = 6e‐06), indicating that patients in cluster 1 could gain greater efficacy in immunotherapy. We used the SubMap algorithm to verify the similarity of expression between TCGA and three immunotherapy queues (GSE78220 (*n* = 28), GSE91061 (*n* = 109) and IMvigor210 (*n* = 348)). The results showed that cluster 1 predicted better immune response in the three queues (Figure S4A‐C).

**FIGURE 2:**
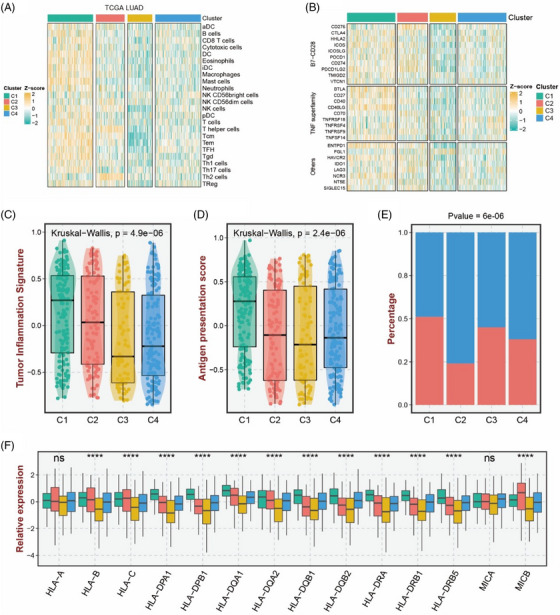
Immune landscape and immune response of each subtype. (A) The heat map of immune cell infiltration of each cluster. (B) The heat map of expression of 27 immune checkpoints in each cluster. (C) Points and boxplots showed the summary statistics and individual values of TIS scores. (D, E) APS scores in each cluster. (F) TIDE scores in each cluster.

We also screened specific drugs for each subtype and evaluated the sensitivity of each subtype to star drugs. As shown in Figure 3A, AS‐703026 obtained from PRISM database can be considered as a specific drug for cluster 1. The specific drugs from the Cancer Therapeutics Response Portal (CTRP) and PRISM databases of cluster 2 are shown in Figure 3B, and the specific drugs of cluster 3 are shown in Figure 3C and Table S3. Then we screened out the area under the curve (AUC) of each subtype against several star drugs; the results are shown in Figure 3D‐H. For example, Cluster 1 was more sensitive to osimertinib and sorafenib.

**FIGURE 3:**
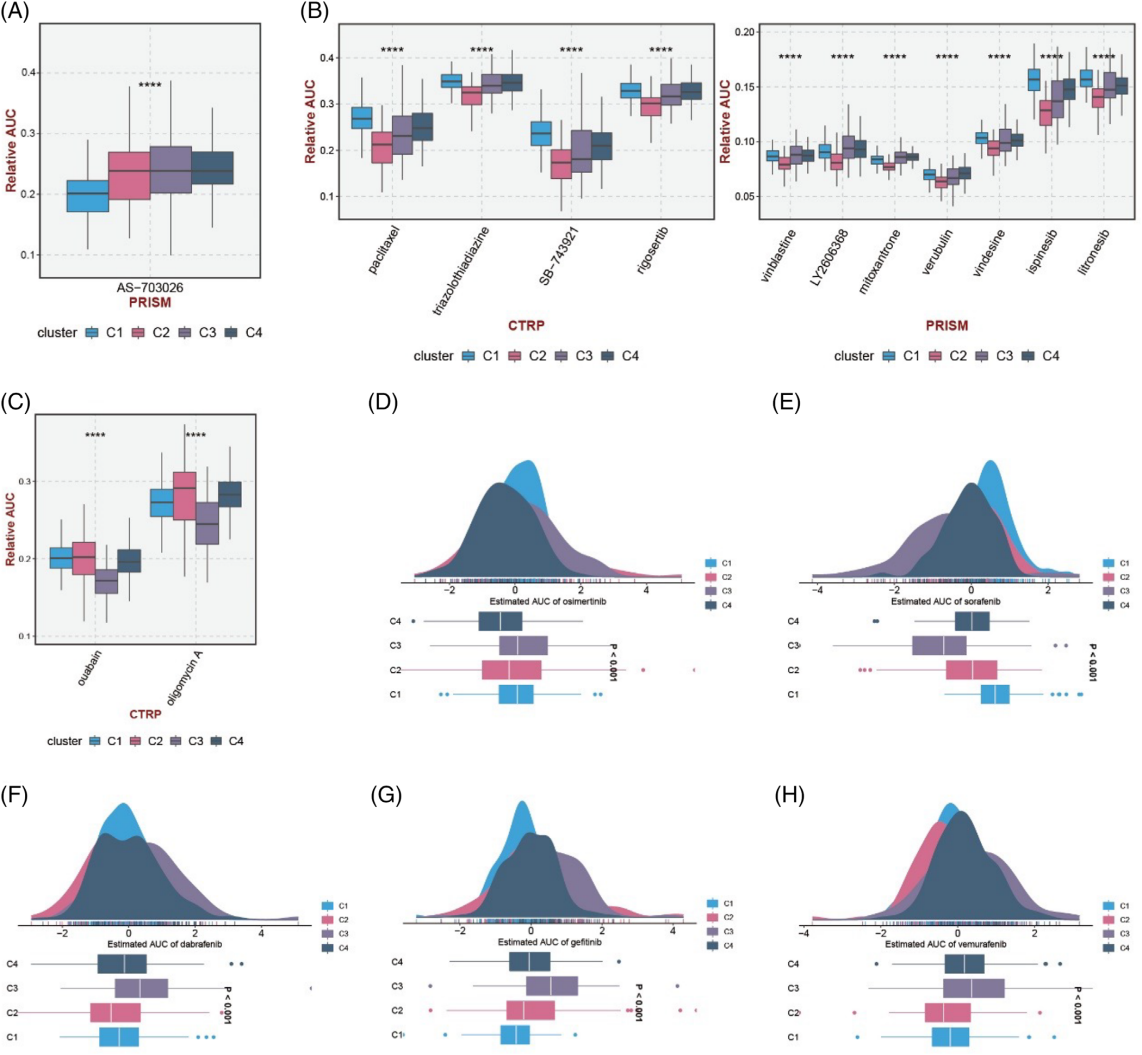
Specific drugs of each subtype and sensitivity of each subtype to star drugs. (A) Sensitive drugs of cluster 1 screened from PRISM database. (B) Sensitive drugs from several cluster 2 of CTRP database and PRISM database. (C) Drugs sensitive to cluster 3 from PRISM database. (D–H) Estimated AUC of osimertinib, solafenib, dabrafenib, gefitinib, vermurafenib in each cluster. Cluster 1 was more sensitive to osimertinib and sorafenib. Cluster 2 was relatively sensitive to sorafenib. Cluster 3 was sensitive to all drugs except sorafenib. Cluster 4 was relatively sensitive to sorafenib and vemurafenib.

We further constructed a GABA‐driven LUAD prognostic signature (GDLPS) based on the above 108 GABA differential genes. Our GDLPS divided LUAD into high‐ and low‐risk group. High‐risk group predicted worse survival outcomes (Figure 4A) (*p* < .0001). Then the GDLPS was verified in three GEO cohorts (GSE31210, GSE41271 and GSE72094), and the results were same as the above results (Figure S5A‐C) (all *p* < .002). We further explored the immune landscape of patients in two groups. Our results displayed that the immune cells infiltration and the immune checkpoints expression of the low‐risk group were higher (Figure 4B and C). The TIS and APS results (Figure 4D and E) showed that the immunotherapy benefits of low‐risk group were higher (*p* = .0059, *p* = 7.2e‐10). Meanwhile, the immunotherapeutic potential of patients was evaluated in three GEO validation queues (Figure S5D‐F), which verified the above calculation results (GSE35640, *p* = .028; GSE91061, *p* = .0027; GSE100797, *p* = .045). Finally, we combined some traditional clinical indicators such as age and stage to build a nomogram (Figure 4F). The sum of the scores obtained from the three indicators is the total score of the patients, which is used to predict the survival probability. The receiver operating characteristic (ROC) curve and calibration plot showed that our nomograph has high accuracy and stability (Figure S5G‐H).

**FIGURE 4:**
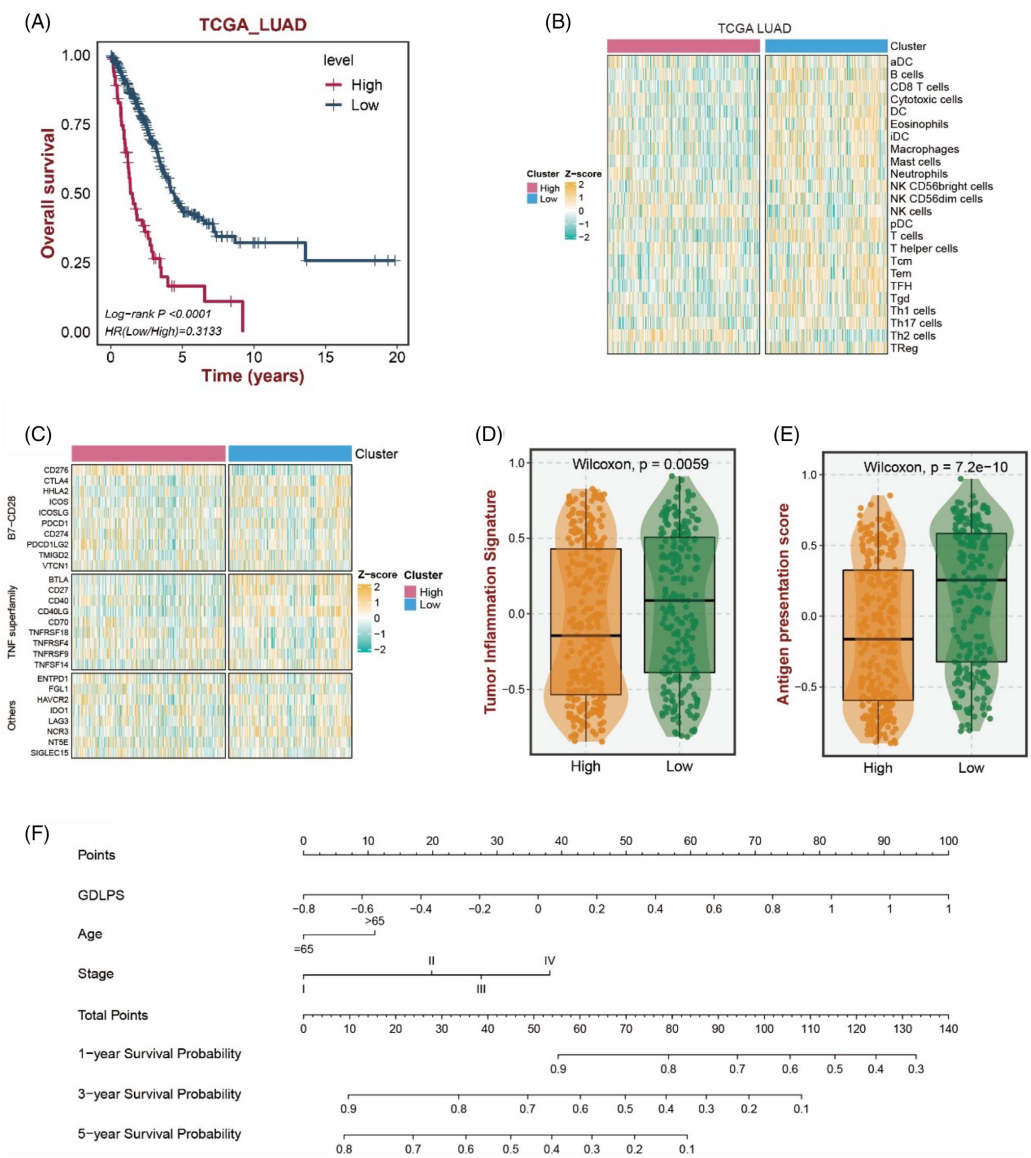
GABA‐driven LUAD prognostic signature for LUAD patients. (A) OS of two groups in TCGA‐LUAD prognosis model. (B) Heat maps of immune cells infiltration abundance in patients with different risk groups. (C) Heat maps of immune checkpoint expression in patients with different risks. (D) TIS scores of the two groups for immunotherapy. (E) APS scores of the two groups for immunotherapy. (F) Nomogram for LUAD patients.

In conclusion, based on the differential expression of GABA in LUAD patients, we classified LUAD patients and comprehensively considered the clinical significance of the classification. We found that differential expression of GABA predicts different prognosis of LUAD. Our study indicated that the different expression of GABA is closely related to immune cell infiltration, immune checkpoint expression and sensitivity to chemotherapy drugs in LUAD patients. We also developed a GABA‐driven prognosis signature for LUAD. Overall, our research indicated that GABA may be an excellent biomarker for evaluating the classification, treatment and prognostic evaluation of patients with LUAD.

## CONFLICT OF INTEREST STATEMENT

The authors declare no potential conflicts of interest.

## FUNDING INFORMATION

The Collaborative Innovation Major Project of Zhengzhou (Grant No. 20XTZX08017), the National Natural Science Foundation of China (Grant No. 82002433), Science and Technology Project of Henan Provincial Department of Education (Grant No. 21A320036), Young and Middle‐aged Health Science and Technology Innovation Talents in 2020(Grant No. YXKC2020049), Henan Province Medical Science and Technology Research Project Joint Construction Project (Grant No. LHGJ20190003, LHGJ20190055).

## Supporting information

Figure S1. The expression of GABA core genes in each subtype. GABA‐related genes were significantly overexpressed in cluster 1. However, the expression of these genes was generally lower in cluster 3.
Figure S2. Comparison of OS between cluster 2 and non‐cluster 2 (A) Comparison of OS between cluster 2 and non‐cluster 2 in TCGA. (B‐D) OS of cluster 2 and non‐cluster 2 in the three GSE cohorts.
Figure S3. Functional enrichment of each subtype. (A‐D) Enrichment score of four clusters.
Figure S4. SubMap analysis of each cluster in three verification cohorts.
Figure S5. (A‐C) OS of two risk groups in three GEO verification queues. (D‐F) Immune response of two groups of patients in three validation queues. (G, H) The ROC curve and calibration plot of nomograph.

Supporting Information

Supporting Information

Supporting Information

Supporting Information

## Data Availability

All datasets generated for this study are included in the article/Supplementary Material.
