# Supplementary material for: Identification and validation of GABA‐driven subtypes and prognosis signature of lung adenocarcinoma
Source: Clin Transl Med. 2023 Oct 19;13(10):e1450. doi: 10.1002/ctm2.1450 (PMC10585196; doi:10.1002/ctm2.1450)
Supplement: Supplementary file 1 — Figure S1. The expression of GABA core genes in each subtype. GABA‐related genes were significantly overexpressed in cluster 1. However, the expression of these genes was generally lower in cluster 3. Figure S2. Comparison of OS between cluster 2 and non‐cluster 2 (A) Comparison of OS between cluster 2 and non‐cluster 2 in TCGA. (B‐D) OS of cluster 2 and non‐cluster 2 in the three GSE cohorts. Figure S3. Functional enrichment of each subtype. (A‐D) Enrichment score of four clusters. Figure S4. SubMap analysis of each cluster in three verification cohorts. Figure S5. (A‐C) OS of two risk groups in three GEO verification queues. (D‐F) Immune response of two groups of patients in three validation queues. (G, H) The ROC curve and calibration plot of nomograph. [file CTM2-13-e1450-s003.docx]

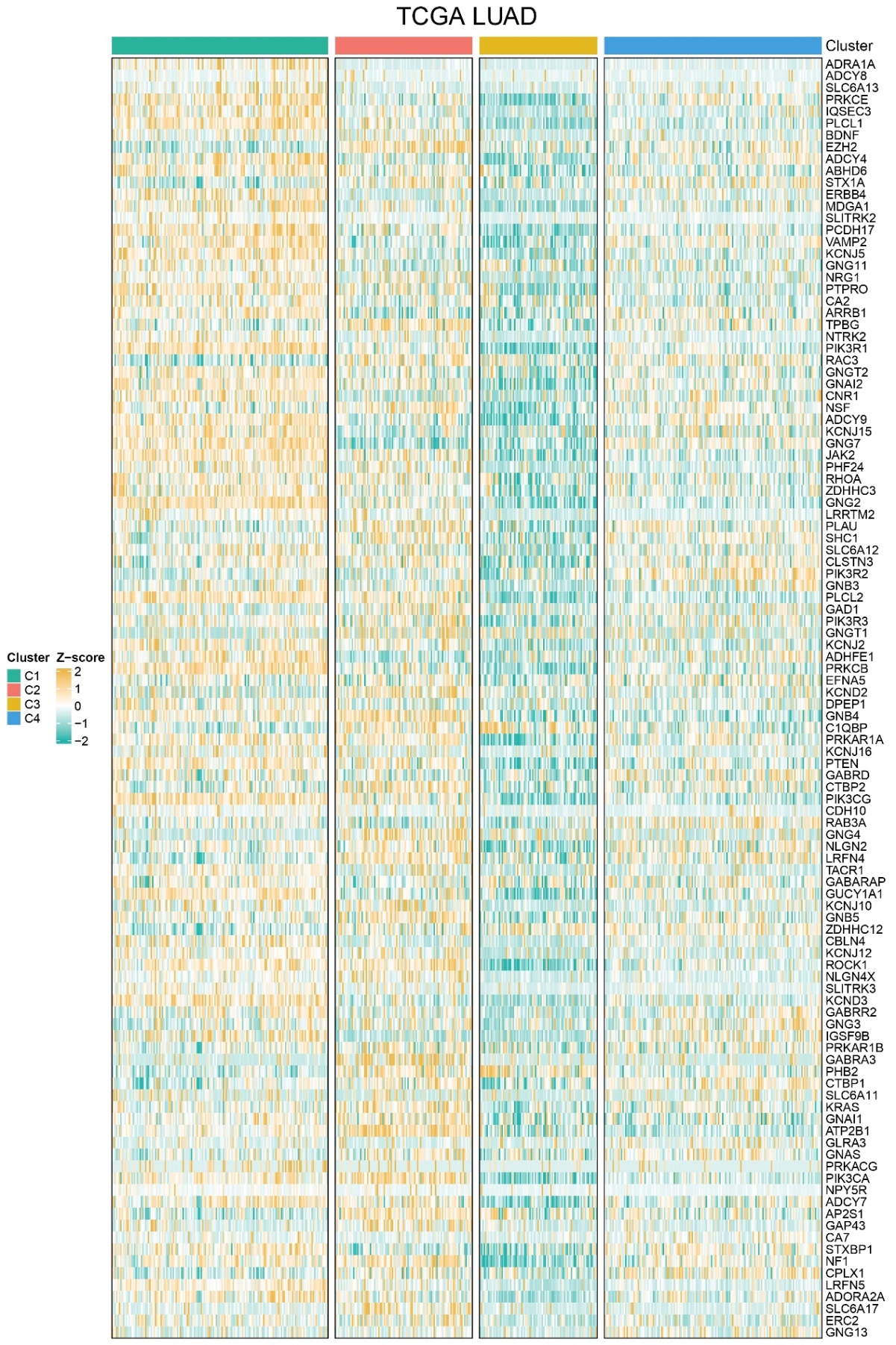


**Figure S1. The expression of GABA core genes in each subtype.** GABA-related genes were significantly overexpressed in cluster 1. However, the expression of these genes was generally lower in cluster 3.


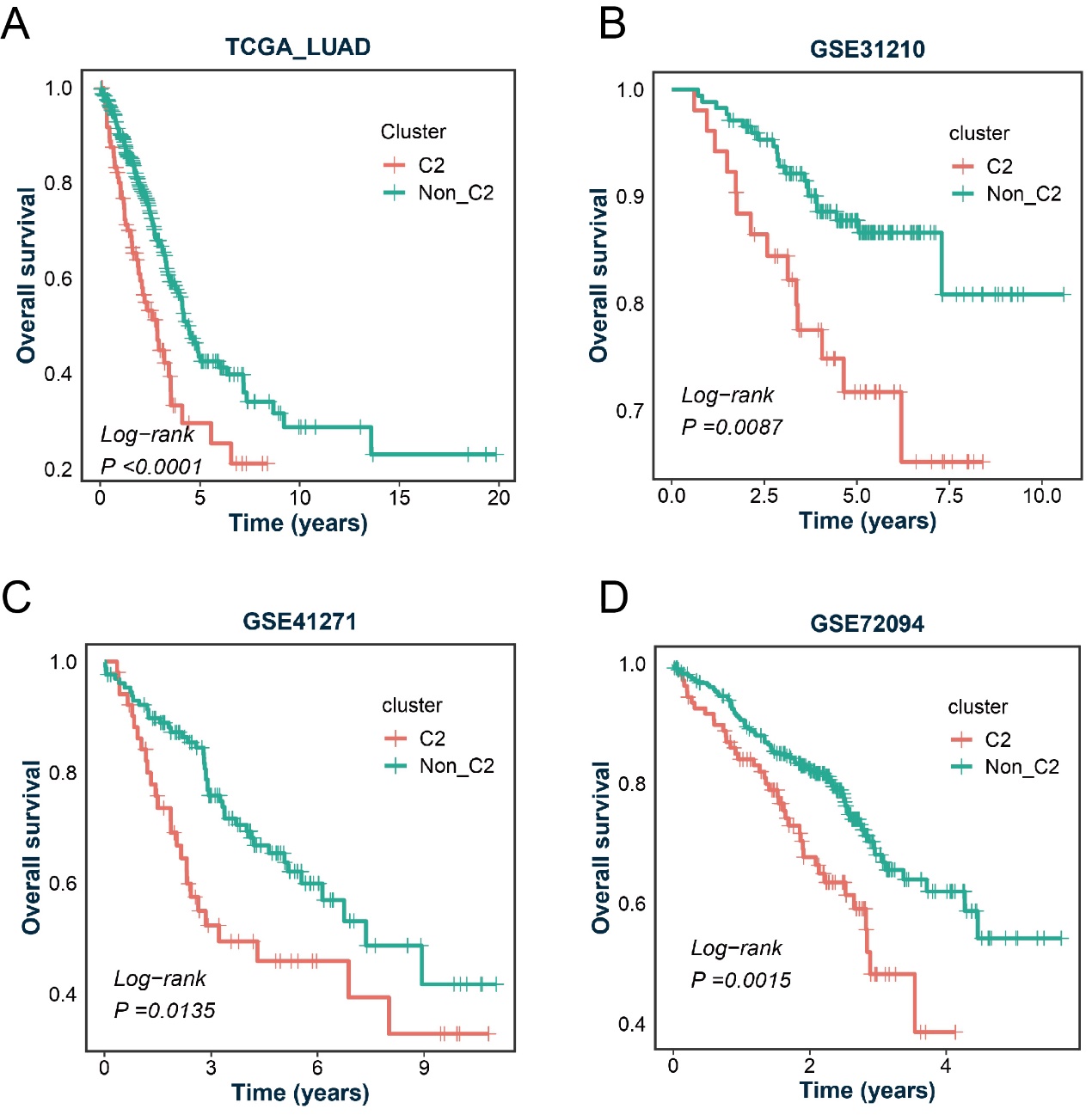


**Figure S2.** **Comparison of OS between cluster 2 and non-cluster 2 (A)** Comparison of OS between cluster 2 and non-cluster 2 in TCGA. **(C-D)** OS of cluster 2 and non-cluster 2 in the three GSE cohorts.


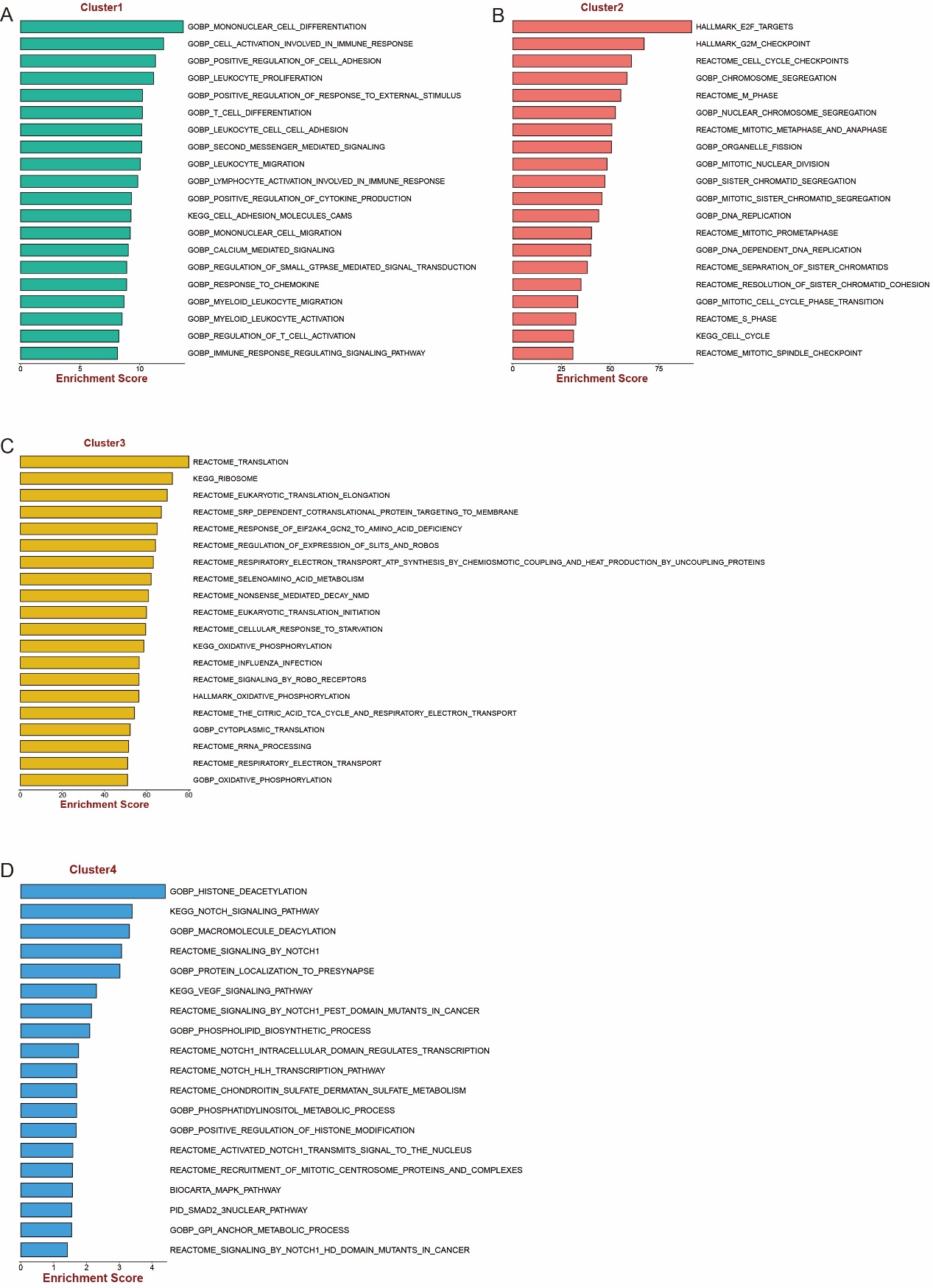


**Figure S3. Functional enrichment of each subtype. (A-D)** Enrichment score of four clusters.


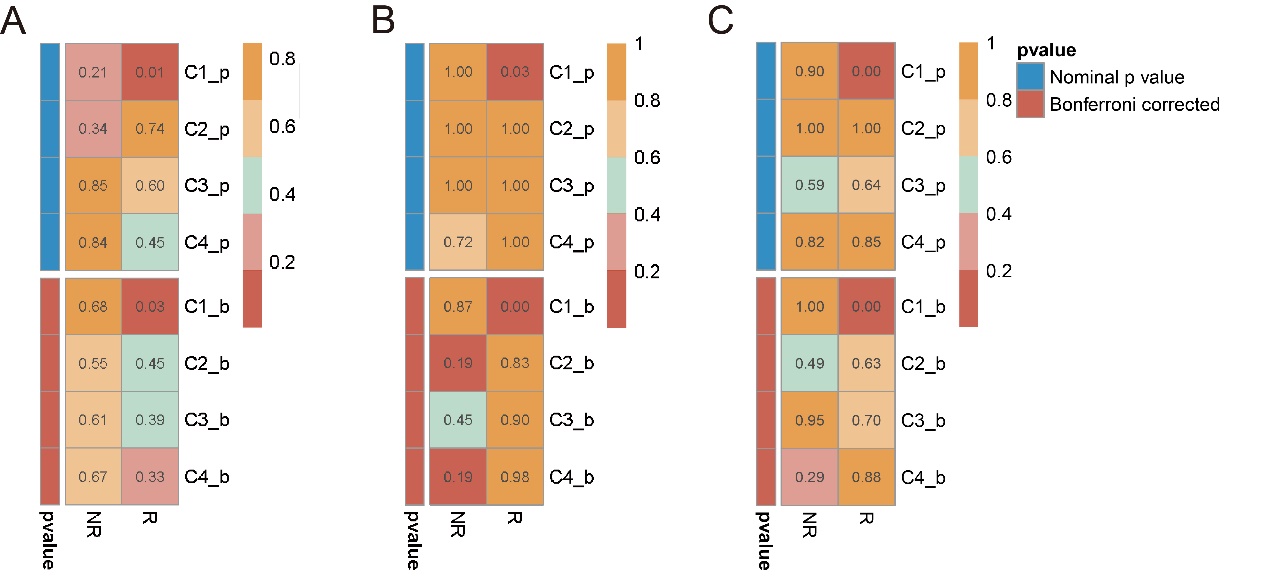


**Figure S4. SubMap analysis of each cluster in three verification cohorts.**

**
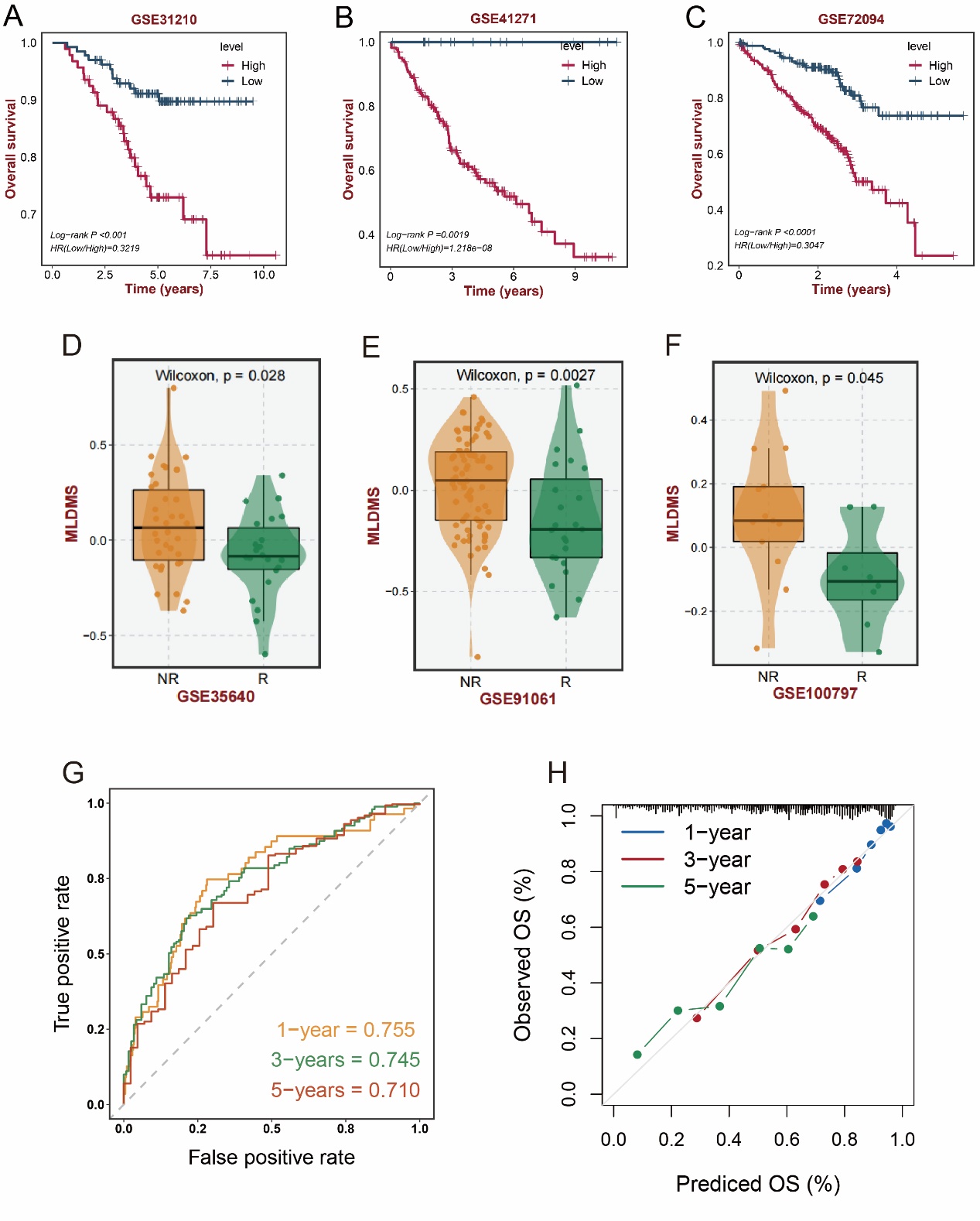
**

**Figure S5.** **(A-C)** OS of two risk groups in three GEO verification queues. **(D-F)** Immune response of two groups of patients in three validation queues. **(G, H)** The ROC curve and calibration plot of nomograph.
