# Supplementary material for: Identification and validation of GABA‐driven subtypes and prognosis signature of lung adenocarcinoma
Source: Clin Transl Med. 2023 Oct 19;13(10):e1450. doi: 10.1002/ctm2.1450 (PMC10585196; doi:10.1002/ctm2.1450)
Supplement: Supplementary file 5 — Supporting Information [file CTM2-13-e1450-s004.docx]

**METHODS**

**Data collection**

Considering the extrapolation of the study and the stability of the results, only LUAD queues with n>100 were retained, and then LUAD sample screening was conducted according to the following criterion conditions: (1) samples were primary tumor tissue; (2) no preoperative radiotherapy or chemotherapy; (3) there were survival materials; (4) there were RNA expression information. Finally, 1361 LUAD samples were obtained, including: TCGA-LUAD (n=555) was downloaded from TCGA database and GSE31210 (n=226), GSE41271 (n=182), GSE72094 (n=398) were downloaded from GEO database; immunotherapy cohort: GSE78220 (n=28), GSE91061 (n=109) were downloaded from GEO database, and IMvigor210 (n=348) was from *Nature* published articles^23^; methylation data and somatic variants data were collected from TCGA and GDC database; Copy number variations data processed based on Genetic Identification of Significant Targets in Cancer 2.0 (GISTIC2.0) algorithm is downloaded from FireBrowse website.

**Construction and verification of GABA driven LUAD subtype**

A comprehensive search for GABA related pathways was conducted from the Mobile Signatures Database (MSigDB) database to obtain GABA related genes. Further, difference analysis of these genes in tumor and normal tissue by “limma” package, core differential genes of GABA were screened out according to the absolute value of logFC>0.5. Based on GABA core differential genes, consensus clustering was conducted in TCGA-LUAD queue using “ConsensusClusterPlus” package. The number of clusters was determined by heat map and delta diagram. Kaplan–Meier analysis was used to predict the overall survival (OS) of each cluster. Then, “Limma” was used to perform difference analysis in TCGA cohort (for example, when analyzing C1 difference, C1 is a group, and the rest subtypes are other groups), and the first 500 differential genes are selected as the characteristic genes of this subtype after being arranged in reverse order according to logFC. The first 500 are selected to avoid over fitting caused by too many genes and insufficient fitting caused by too few genes. Based on the characteristic genes of each subtype, the samples were classified in three GEO queues (GSE31210, GSE41271, GSE72094) using Nearest Template Prediction (NTP) algorithm^24^. Then, OS of three GEO queues as verifications was predicted by using Kaplan–Meier analysis. Then use the Submap algorithm to compare the similarity between the training set TCGA and the three GEO verification sets, to further verify the subtypes.

**Functional Enrichment Analysis**

9570 gene set pathways were collected from the MSigDB database, including 50 cancer hallmark gene sets, 186 Kyoto Encyclopedia of Genes and Genomes (KEGG) gene sets, 196 Pathway Interaction Database (PID) gene sets, 289 Biocarta gene sets, 1499 Reactome gene sets and 7350 GO biological process gene sets. In this study, the “clusterProfiler” package in R software was used to perform the functional enrichment analysis of four subtypes in LUAD.

**Immune landscape and immune responds**

The single-sample gene set enrichment analysis (ssGSEA) algorithm was used to evaluate the infiltration abundance of 24 kinds of immune cells in each subtype sample of TCGA-LUAD queue. Then the same method was used to evaluate the expression difference of 27 immune checkpoints in each subtype. Then the Tumor Inflammation Signature (TIS), Antigen presentation score (APS), Tumor Immune Dysfunction and Exclusion (TIDE) algorithms were used to evaluate the response of each subtype to immunotherapy. And Submap algorithm was used to verify TCGA training set in three verifications set, including GSE78220 and GSE91061 from GEO database, and IMvigor210 from published articles of Nature^23^. The same method was used to predict the immune cell infiltration, immune checkpoint expression and immune response of patients with different risk groups in the later prognosis model.

**Screening of specific drugs**

The gene expression and drug sensitivity data of hundreds of cell lines were obtained from CTRP and PRISM websites respectively. After removing samples with deletion values of more than 20% and cell lines from hematopoietic and lymphoid tissues, 266 and 1285 compounds were obtained from CTRP and SCR_005375 databases respectively. Then, according to the expression profile, “pRRophic” package based on ridge regression algorithm is used to predict drug sensitivity, so as to obtain the AUC value of each compound in the sample. After that, we analyzed the difference of AUC of each drug in patients with different subtypes, and then screened out the sensitive drugs of specific subtypes. Specifically, when screening C1 subtype sensitive drugs, if the logFC of C2, C3, C4 and C1 subtype AUC is greater than 0.1, the drug was considered as a sensitive drug of C1 subtype.

**Establishment of GABA based prognostic signature for LUAD**

Univariate Cox regression analysis was performed on 108 differential genes, and differential genes with prognostic significance (p<0.05) were screened out. Based on these genes, ridge regression algorithm (Ridge) was used to construct prognostic model in TCGA. The OS of model was obtained via Kaplan–Meier analysis. Considering the clinical practicability and combining with the traditional clinical indicators, the nomograms of 1-year, 3-year and 5-year survival probability prediction were developed. Receiver operating characteristic (ROC) curve analysis.

**Immunohistochemical (IHC)**

We collected pathological sections of 12 LUAD patients and divided them into two groups according to whether they survived for 1 year after surgery: good prognosis and poor prognosis. The slices were placed in a container containing antigen recovery solution, and then the container was placed in a microwave oven. The container was heated to a boil on high heat for 2 minutes, followed by a medium and low heat for 20 minutes. The container containing the antigen repair solution and sections was removed from the microwave and allowed to cool in a room temperature environment. Then endogenous peroxidase was inactivated by incubating with 3% hydrogen peroxide diluted in methanol for 10min at room temperature, followed by washing with PBS solution for 3 times, 5min each time. The sections were removed and wiped with clean gauze to remove residual PBS. The sections were subsequently placed in a box with a moist environment. Reagent A was added to the wet box and incubation was carried out for 30min at room temperature. Subsequently, about 60μl of primary antibody was added dropfold into a wet box, which was subsequently placed in a 4 ° C refrigerator and incubated. The wet box was removed from the refrigerator and placed in a room temperature environment for 1h. Then the cells were washed with PBS for a total of 4 times, 5min each time. Then the secondary antibody working solution was added and incubated at room temperature for 0.5h, and then washed once with PBS solution for a total of 5min. Then reagent C was added to the wet box, and the mixture was incubated at room temperature for 15 min, followed by PBS washing for 3 times, 5min each time. DAB liquid was added to the section for color development, and the color development was stopped when light brown appeared. Rinse with running water to ensure complete termination of color development. The slices were wiped with clean gauze and then placed in a wet box, followed by the addition of hematoxylin. After 15min, the slices were rinsed with running water. The sections were immersed in gradient alcohol solutions for 1min each, followed by 5min in each of the two xylene solutions. After removal, the pieces were sealed. EZH2 antibody (**Cat No:** 66476-1-Ig) was purchased from Proteintech Biological Inc.

**Statistical Analysis**

In this study, all of the data were analyzed by R software (4.1.0). The survival data between different groups were compared by log-rank test, p < 0.05 was considered as statistically significant.
